# Microsatellite distribution on sex chromosomes at different stages of heteromorphism and heterochromatinization in two lizard species (Squamata: Eublepharidae: *Coleonyx elegans *and Lacertidae: *Eremias velox*)

**DOI:** 10.1186/1471-2156-12-90

**Published:** 2011-10-20

**Authors:** Martina Pokorná, Lukáš Kratochvíl, Eduard Kejnovský

**Affiliations:** 1 Department of Ecology, Faculty of Science, Charles University in Prague, Viničná 7, 128 44 Praha 2, Czech Republic; 2 Department of Vertebrate Evolutionary Biology and Genetics, Institute of Animal Physiology and Genetics, Academy of Sciences of the Czech Republic, Rumburská 89, 277 21 Liběchov, Czech Republic; 3 Laboratory of Plant Developmental Genetics, Institute of Biophysics, Academy of Sciences of the Czech Republic, Královopolská 135, 612 65 Brno, Czech Republic

## Abstract

**Background:**

The accumulation of repetitive sequences such as microsatellites during the differentiation of sex chromosomes has not been studied in most squamate reptiles (lizards, amphisbaenians and snakes), a group which has a large diversity of sex determining systems. It is known that the *Bkm *repeats containing tandem arrays of GATA tetranucleotides are highly accumulated on the degenerated W chromosomes in advanced snakes. Similar, potentially homologous, repetitive sequences were found on sex chromosomes in other vertebrates. Using FISH with probes containing all possible mono-, di-, and tri-nucleotide sequences and GATA, we studied the genome distribution of microsatellite repeats on sex chromosomes in two lizard species (the gecko *Coleonyx elegans *and the lacertid *Eremias velox*) with independently evolved sex chromosomes. The gecko possesses heteromorphic euchromatic sex chromosomes, while sex chromosomes in the lacertid are homomorphic and the W chromosome is highly heterochromatic. Our aim was to test whether microsatellite distribution on sex chromosomes corresponds to the stage of their heteromorphism or heterochromatinization. Moreover, because the lizards lie phylogenetically between snakes and other vertebrates with the *Bkm*-related repeats on sex chromosomes, the knowledge of their repetitive sequence is informative for the determination of conserved versus convergently evolved repetitive sequences across vertebrate lineages.

**Results:**

Heteromorphic sex chromosomes of *C. elegans *do not show any sign of microsatellite accumulation. On the other hand, in *E. velox*, certain microsatellite sequences are extensively accumulated over the whole length or parts of the W chromosome, while others, including GATA, are absent on this heterochromatinized sex chromosome.

**Conclusion:**

The accumulation of microsatellite repeats corresponds to the stage of heterochromatinization of sex chromosomes rather than to their heteromorphism. The lack of GATA repeats on the sex chromosomes of both lizards suggests that the *Bkm*-related repeats on sex chromosomes in snakes and other vertebrates evolved convergently. The comparison of microsatellite sequences accumulated on sex chromosomes in *E. velox *and in other eukaryotic organisms suggests that historical contingency, not characteristics of particular sequences, plays a major role in the determination of which microsatellite sequence is accumulated on the sex chromosomes in a particular lineage.

## Background

The evolution of sex chromosomes from autosomes has been documented many times in different organisms [1]; recently reviewed e.g. in [2]; but see e.g. [3]]. During their evolution, sex chromosomes go progressively through several steps. Briefly, the first step is the acquisition of sex determining locus or loci. Subsequently, the genetic content of both members of the pair diverge. The specialization of sex chromosomes for their sex-specific roles [e.g. [4]] selects for the reduction of the interchange of genetic material between sex chromosomes and thus for lower levels of recombination. However, lack of recombination leaves the unpaired sex chromosomes (Y and W) without the possibility to correct mutations in coding sequences, which leads to an unusually low content of functional genes. Moreover, cessation of recombination opens doors for the accumulation of various repeats on sex chromosomes (e.g. microsatellites, transposons, rDNA sequences; [5]). Alternatively, the accumulation of repetitive sequences may not be a consequence of reduced recombination, but its cause [6]. By generating asynchrony in the DNA replication pattern of X and Y, respectively Z and W chromosomes, it can reduce the crossing-over frequency between them [e.g. [7]]. The accumulation of repeats on a heterogametic sex chromosome (Y or W) may be so massive that the chromosome is finally much larger than its homologous counterpart in the pair. The heterogametic sex chromosome may even become the largest chromosome in the genome such as the Y chromosome in the plant *Silene latifolia *[[8], [9]]. On the other hand, in some lineages, heterogametic sex chromosomes may progressively decrease in size [e.g. [10]] and such degeneration can result in their elimination from the genome [e.g. [11]]. In yet other cases, sex chromosomes may stay homomorphic for a long evolutionary time [e.g. [10,12,13]]. In many organisms, the heterogametic sex chromosome has been found to be highly heterochromatinized [e.g. [12,14]]. The heterochromatinization may be a mechanism for the defence against the activity of transposable elements or other repetitive sequences to safeguard genome integrity [e.g. [15,16]].

Squamate reptiles, the lineage encompassing lizards, snakes and amphisbaenians, represent an interesting group for the exploration of the evolution of sex chromosomes, as they possess substantial variability in sex determining mechanisms [17-19]. Squamate reptiles include species with environmental sex determination, i.e. without sex chromosomes; species with homomorphic sex chromosomes, and those with heteromorphic sex chromosomes. All three situations can be found even in a single family, for example in dragon lizards or eye-lid geckos [20-22]. Sex chromosomes are at various stages of the general process of sex chromosome evolution in different squamate species and they evolved within squamates independently several times as supported by differences in their size, shape and type (male or female heterogamety) but also by molecular-cytogenetic tests of synteny of sex chromosomes and phylogenetic distribution of sex determining systems [10,19,20,23].

At the time of submitting, to our knowledge, the accumulation of repeats during the degeneration of sex chromosomes has been studied only in a single lineage of squamate reptiles, in that of snakes [7,24,25]. Pythons, the group in the rather basal position of snake phylogeny [26], with homomorphic sex chromosomes, do not show any accumulation of repeats, while the degenerated W sex chromosomes in many advanced snakes from the crown clade Colubroidea such as colubrids or elapids, exhibit a massive accumulation of repeats [24,25]. For example, the W chromosome in an elapid snake *Notechis scutatus *is composed almost entirely of repetitive sequences, including 18S rDNA and the banded krait minor-satellite (*Bkm*) repeats [26]. The *Bkm *repeats consist of tandem arrays of 26 and 12 copies, respectively, of two tetranucleotides, GATA and GACA [27]. *Bkm*-related repeats are also accumulated on the heterogametic sex chromosomes in many vertebrates, including humans, and also in plants [28-33]. It was speculated that the *Bkm*-related repeats are functional, playing a role in the transcriptional activation of sex chromosome heterochromatin [7]. A common origin of the *Bkm*-related repeats across different eukaryotic lineages was assumed [e.g. [34]], however, a convergent evolution is also likely [35]. Recently, based on the results of chicken W chromosome painting in snakes, O'Meally and colleagues [25] concluded that heterogametic sex chromosomes in birds and derived snakes may share repetitive sequences. They suggested that this observation could be explained by yet undetected synteny of parts of the sex chromosomes between these lineages. The homology of repetitive sequences accumulated on sex chromosomes could be tested by the evaluation of the identity of repeats on sex chromosomes in other lineages of squamates phylogenetically nested between snakes, birds and vertebrates with the *Bkm*-related repeats.

The aim of the present study is to compare the distribution of microsatellite sequences on differently differentiated sex chromosomes in two lizard species with independently evolved sex chromosomes and to determine whether the distribution of microsatellite repeats on sex chromosomes corresponds to the stage of their heteromorphism or heterochromatinization. Multiple sex chromosomes (X_1_X_1_X_2_X_2_/X_1_X_2_Y) are heteromorphic and fully euchromatic in the first studied species, the gecko *Coleonyx elegans *from the family Eublepharidae [21]. On the other hand, the ZZ/ZW sex chromosomes in the second species, *Eremias velox *from the family Lacertidae, are homomorphic and the W chromosome is highly heterochromatic [36]. Moreover, the gekkotan lizards represent one of the basal groups of squamate reptiles, while lacertids are much more closely related to snakes [37]. The knowledge of repetitive sequences on the sex chromosomes in the two selected species should therefore be informative for the determination of the homology of the *Bkm*-related and other repeats across vertebrate lineages.

## Methods

The lizard individuals involved in the study were captive bred animals maintained in the breeding room at the Faculty of Science, Charles University in Prague, Czech Republic (accreditation No. 24773/2008-10001). The procedures on animals were held under the approval and supervision of the Ethical Committee of the Faculty of Science, Charles University in Prague (permission No. 29555/2006-30). Metaphase chromosome spreads were prepared from cultures of whole blood from a male of *C. elegans *and a female of *E. velox *following the protocols described by Ezaz and colleagues [14] with slight modifications. C-banding was performed following the method described by Pokorná and colleagues [21].

Oligonucleotides containing microsatellite sequences were directly labelled with Cy3 at the 5' end during synthesis by VBC-Biotech (Wien, Austria). All possible mono- (d(A)_30_, d(C)_30_), di- (d(CA)_15_, d(GA)_15_, d(GC)_15_, d(TA)_15_), and tri-nucleotides (d(CAA)_10_, d(CAG)_10_, d(CGG)_10_, d(GAA)_10_, d(CAC)_10_, d(CAT)_10_, d(GAC)_10_, d(GAG)_10_, d(TAA)_10_, d(TAC)_10_) and d(GATA)_8 _were used. The tetranucleotide was included to test for the presence of the *Bkm*-related repeats. Slide denaturation was performed in 7:3 (v/v) formamide:2xSSC for two minutes at 72°C, then the slides were dehydrated using 50%, 70% and 100% ethanol (-20°C) serie and air-dried. The probes were denatured at 70°C for 10 minutes in a mix containing 50% formamide (v/v), 2xSSC and 10% dextransulfate (w/v) and subsequently applied to the slides, covered with plastic coverslips, and hybridized for 18 hours at 37°C. The slides were washed at room temperature twice for 5 minutes in 2xSSC and twice for 5 minutes in 1xSSC. The slides were analysed using an Olympus Provis microscope and the image analysis was performed using ISIS software (Metasystems). The FISH results were confirmed in at least three different metaphases per treatment.

The heterogametic sex chromosomes in both studied species of lizards are easily recognizable. The Y chromosome is the largest and the only metacentric chromosome in karyotype of *C. elegans *[21]. The W chromosome of *E. velox *is an acrocentric chromosome of similar size to the Z chromosome, but only the W is conspicuously DAPI-positive [23].

## Results

There was no accumulation of mono-, di-, tri-nucleotides or GATA repeats detected on sex chromosomes in *C. elegans*. All tested sequences showed relatively uniform distribution throughout the genome of this species.

**Figure 1:**
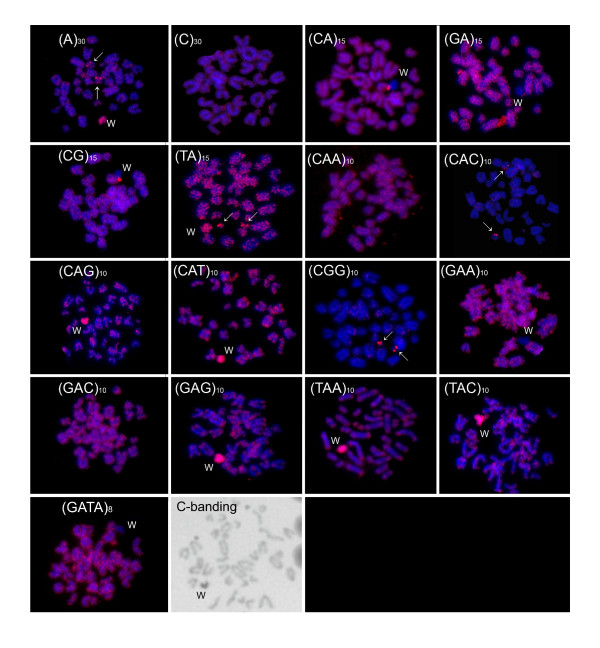
**Mitotic metaphase chromosomes of *Eremias velox *females hybridized with different microsatellite-containing oligonucleotides**. Chromosomes were counterstained with DAPI (blue) and microsatellite probes were labelled with Cy3 (red signals). Last figure represents C-banded metaphase chromosomes. Letters mark the W chromosomes, arrows indicate autosomal signals.

Strong accumulations of several microsatellites were detected either on the W chromosome or on some autosomes in *E. velox *(Figure 1). The W chromosome showed an interspersed distribution pattern of microsatellite sequences typical for the Z chromosome and autosomes only in three cases, i.e. in the probes d(C)_30_, d(CAA)_10 _and d(GAC)_10_. The d(CGG)_10 _and d(CAC)_10 _sequences exhibited notable accumulations just on small autosomes. Only in two cases (d(A)_30_, d(TA)_15_) was there a concurrently notable accumulation of microsatellites on the whole W chromosome and on two different pairs of autosomes. In all other cases, the W chromosome showed a more highly distinct pattern than the Z chromosome and all autosomal pairs. Some microsatellites with tri-nucleotide motifs (d(CAG)_10_, d(CAT)_10_, d(GAG)_10_, d(TAC)_10_, d(TAA)_10_) were extensively accumulated over the whole length of the W chromosome, while three di-nucleotide repeats (d(CA)_15_, d(GA)_15_, d(GC)_15_) were accumulated just in the centromeric parts of the W chromosome. Three microsatellite sequences (d(GA)_15_, d(GAA)_10_, d(GATA)_8_) were conspicuously lacking on the W chromosome, although the signal was otherwise uniformly distributed across the rest of the genome.

## Discussion

The FISH patterns using the probes bearing microsatellite repeats in the two lizard species contrasted greatly. The FISH experiments did not reveal a substantial accumulation of microsatellites neither on X_1_, X_2 _and Y sex chromosomes nor on autosomes in the gecko *C*. *elegans*. The Y chromosome is the largest chromosome in the karyotype and certain parts of the Y and the X_1 _chromosome consist largely of repetitive elements. Specifically, both these chromosomes carry notable accumulations of 28S rDNA repeats [Figs. two d, i in [21]]. Nevertheless, these accumulations are restricted to just nucleolus organizer regions (NORs), although rDNA-related repeats have the potential to spread over sex chromosomes [38]. Previously, through chromosome painting, we documented that the Y and X_1 _and X_2 _chromosomes in *C. elegans *share similar DNA content and concluded that sex chromosomes of this species are only poorly differentiated [21]. The results of the painting with the microsatellite probes further support this conclusion.

The heterochromatinized W chromosome and some autosomes show strong microsatellite accumulation in the lacertid lizard *E. velox *(Figure 1). The distribution of microsatellite sequences on the W chromosome differed greatly in various microsatellites. We found enrichment of some microsatellite sequences over the whole W chromosome, while the accumulation of some sequences (CA, GA, and GC repeats) was restricted to a part of the W chromosome near the centromere. This unequal distribution could reflect constitutional characteristics of individual microsatellites, e.g. their convenience for centromere formation; however, no accumulation of CA, GA, and GC repetitive sequences was detected in the centromeres of the Z chromosome and the autosomes (Figure 1). Alternatively, the unequal distribution could reflect an instantaneous stage of competition of individual microsatellite sequences over a limited number of positions on the W chromosome. Some sequences are present on the Z chromosome and autosomes, but they are notably lacking on the W chromosome (Figure 1). Generally, sex chromosomes at the earliest stages of differentiation mutually differ only in a small non-recombining sex-determining region and are otherwise similar to autosomes [e.g.[4]]. Therefore, it seems likely that the comparable distribution of these sequences on the recent Z chromosome and on autosomes in *E. velox *reflects the ancestral situation and that the sequences were present on the W chromosome at an early stage of its differentiation as well and were later replaced by more successful sequences. The alternative explanations on unequal distribution of particular repeats can be tested by the reconstruction of evolutionary dynamics of microsatellite distribution on sex chromosomes across lacertids in future comparative analyses.

The microsatellite distribution in *C. elegans *and *E. velox *corresponds to the general scenario of sex chromosome evolution. Sex chromosomes in *C. elegans*, although heteromorphic, probably represent an early stage of sex chromosome differentiation. The lack of microsatellite accumulation on sex chromosomes in this species is comparable to the situation on homomorphic sex chromosomes in relatively basal snakes [7,25]. The present study documents that the DNA content strongly differs between the euchromatic Z and the heterochromatic W chromosomes in *E. velox *(Figure 1), although the sex chromosomes are homomorphic in this species and in many other lacertids [12]. Heteromorphic sex chromosomes are not always simply more diverged than homomorphic ones. The changes of DNA sequences and of karyotype during the evolution of sex chromosomes can be quite different stories. However, in most cases, the accumulation of microsatellites precedes the evolution of the heteromorphy of sex chromosomes. Heteromorphic sex chromosomes with accumulated repeats, e.g. in the advanced snakes from the families Elapidae and Colubridae [25], may represent only the later stage of the evolution of sex chromosomes.

Members of many genera in the family Lacertidae from both its subfamilies (Gallotiinae and Lacertinae) have the ZZ/ZW sex-chromosome system with sex chromosomes at various stage of differentiation [12,39,40]. Phylogenetic distribution of species with known sex chromosomes suggests that female heterogamety is ancestral for the family [e.g. [19]; *cf*. to phylogenetic relationships within the family in [39] or [41]]. A molecular clock based on mitochondrial DNA sequences indicates that the separation of the Gallotiinae and Lacertinae occurred around 20 My ago [39]. The mechanism keeping homomorphy of sex chromosomes in the lineage leading to *E. velox *and in other lacertids for such a long time in the face of the highly divergent DNA content of sex chromosomes is not known.

The *Bkm-*related repeats containing tandem copies of GATA sequence have been shown to be accumulated on the sex chromosomes of various eukaryotes including advanced snakes [25]. The GATA repeats are uniformly distributed over the autosomes and sex chromosomes in *C. elegans*, and the W chromosome of *E. velox *even exhibits a conspicuous depletion of this sequence (Figure 1), which supports the independent origins of the *Bkm*-related repetitions on sex chromosomes in snakes and other vertebrates. O'Meally and colleagues [25] reasoned that as derived snake and bird sex chromosomes share common repetitive sequences, this may be due to the cryptic homology of parts of the sex chromosomes between these lineages. However, no FISH signal on the W chromosome was observed after hybridization of the *Bkm *probe to chicken metaphase chromosomes in their study. Moreover, sex chromosomes in snakes and birds evolved from different autosomal pairs [10], and basal snake and avian lineages have homomorphic sex chromosomes without an accumulation of repetitive sequences [13,24,25]. The independent origins of repetitive sequences on degenerated W sex chromosomes in both these lineages therefore seem more likely, especially when high dynamism of repetitive DNA is taken into account.

Due to different biochemical characteristics, particular microsatellite sequences should differ in their potency to accumulate on sex chromosomes. We should then observe an accumulation of the same sequences on independently evolved sex chromosomes in different organisms. However, the data accumulated so far does not support this prediction. For example, among all possible trinucleotide sequences, tandem copies of CAA, CAG, GAA and TAA showed the most notable accumulation on the Y chromosome in the plant *Silene latifolia *[42]. However, out of these four sequences, only CAG and TAA tandem copies are accumulated on the W chromosome, while CAA repeats are uniformly distributed across all chromosomes and GAA repeats are even lacking on the W chromosome in *E. velox *(Figure 1). Similarly, the CGG repeats are accumulated on the Y chromosome of the fish *Hoplias malabaricus *[43], but they are missing on the W chromosome of the lizard. In conclusion, various repetitive sequences follow very different trajectories on sex chromosomes in different organismal lineages. The identity of particular microsatellite sequences accumulated on sex chromosomes seems to largely reflect historical contingency.

## Conclusions

In conclusion, our results support the view of sex chromosome evolution as a colourful myriad of situations and trajectories where many processes, often opposing, are in action. The evolution of DNA sequences and the evolution of karyotypes can represent different stories of the shaping of sex chromosomes. Microsatellite dynamics can be tied rather to heterochromatinization than to heteromorphism of sex chromosomes. Microsatellites are the most dynamic component of genomes, and as non-recombining regions of the sex chromosomes give them the chance to expand here, their contribution to sex chromosomes dynamics is significant. It seems that historical contingency, not the characteristics of particular sequences, plays a major role in the determination of which microsatellite sequence is accumulated on the sex chromosomes in a particular lineage.

## Authors' contributions

MP carried out the leukocyte cultures preparation and chromosome preparation and participated on the design of the study and the drafting the manuscript. LK participated on the chromosome preparation, design of the study, and led the drafting of the manuscript. EK carried out FISH experiments, participated on the design of the study and the drafting of the manuscript. All authors have read and approved the final manuscript.

## References

[B1] Ohno S Sex chromosomes and sex-linked genes 1967 Springer-Verlag Berlin. Heidelberg. New York

[B2] Charlesworth D Mank J The birds and the bees and the flowers and the trees: Lessons from genetic mapping of sex determination in plants and animals Genetics 2010 186 9 31 10.1534/genetics.110.117697 20855574 PMC2940314

[B3] Carvalho AB Origin and evolution of the *Drosophila *Y chromosome Curr Opin Genet Dev 2002 12 664 668 10.1016/S0959-437X(02)00356-8 12433579

[B4] Rice WR Sex chromosomes and the evolution of sexual dimorphism Evolution 1984 38 735 742 10.2307/2408385 28555827

[B5] Charlesworth B Novartis Foundation The evolution of chromosomal sex determination Genetics and Biology of Sex Determination 2002 John Wiley and sons Ltd 207 224

[B6] Steinemann S Steinemann M Y chromosomes: born to be destroyed BioEssays 2005 27 1076 1083 10.1002/bies.20288 16163733

[B7] Singh L Purdom IF Jones KW Satellite DNA and evolution of sex chromosomes Chromosoma 1976 59 43 62 10.1007/BF00327708 1001165

[B8] Kejnovsky E Kubat Z Hobza R Lengerova M Sato S Tabata S Fukui K Matsunaga S Vyskot B Accumulation of chloroplast DNA sequences on the Y chromosome of *Silene latifolia* Genetica 2006 128 167 175 10.1007/s10709-005-5701-0 17028949

[B9] Kejnovsky E Hobza R Kubat Z Cermak T Vyskot B The role of repetitive DNA in structure and evolution of sex chromosomes in plants Heredity 2009 102 533 541 10.1038/hdy.2009.17 19277056

[B10] Matsubara K Tarui H Toriba M Yamada K Nishida-Umehara Ch Agata K Matsuda Y Evidence for different origin of sex chromosomes in snakes, birds, and mammals and stepwise differentiation of snake sex chromosomes Proc Natl Acad Sci USA 2006 103 18190 18195 10.1073/pnas.0605274103 17110446 PMC1838728

[B11] Fredga K Aberrant chromosomal sex-determining mechanisms in mammals, with special reference to species with XY females Phil Trans R Soc Lond B 1988 322 83 95 10.1098/rstb.1988.0116 2907806

[B12] Olmo E Odierna G Capriglione T Evolution of sex-chromosomes in lacertid lizards Chromosoma 1987 96 33 38 10.1007/BF00285880

[B13] Tsuda Y Nishida-Umehara C Ishijima J Yamada K Matsuda Y Comparison of the Z and W sex chromosomal architectures in elegant crested tinamou (*Eudromia elegans*) and ostrich (*Struthio camelus*) and the process of sex chromosome differentiation in palaeognathous birds Chromosoma 2007 116 159 173 10.1007/s00412-006-0088-y 17219176

[B14] Ezaz T Quinn AE Miura I Sarre D Georges A Graves JAM The dragon lizard *Pogona vitticeps *has ZZ/ZW micro-sex chromosomes Chromosome Res 2005 13 763 776 10.1007/s10577-005-1010-9 16331408

[B15] Biemont C Are transposable elements simply silenced or are they under house arrest? Trends Genet 2009 25 333 334 10.1016/j.tig.2009.05.006 19577319

[B16] Grewal SIS Jia S Heterochromatin revisited Nat Rev Genet 2007 8 35 46 10.1038/nrg2008 17173056

[B17] Janzen FJ Phillips PC Exploring the evolution of environmental sex determination, especially in reptiles J Evol Biol 2006 19 1775 1784 10.1111/j.1420-9101.2006.01138.x 17040374

[B18] Organ C Janes DE Evolution of sex chromosomes in Sauropsida Integr Comp Biol 2008 48 512 519 10.1093/icb/icn041 21669812 PMC4553705

[B19] Pokorná M Kratochvíl L Phylogeny of sex-dermining mechanisms in squamate reptiles: Are sex chromosomes an evolutionary trap? Zool J Linn Soc 2009 156 168 183 10.1111/j.1096-3642.2008.00481.x

[B20] Ezaz T Quinn AE Sarre SD O'Meally D Georges A Graves JAM Molecular marker suggests rapid changes of sex-determining mechanisms in Australian dragon lizards Chromosome Res 2009 17 91 98 10.1007/s10577-008-9019-5 19172405

[B21] Pokorná M Rábová M Ráb P Ferguson-Smith MA Rens W Kratochvíl L Differentiation of sex chromosomes and karyotypic evolution in the eye-lid geckos (Squamata: Gekkota: Eublepharidae), a group with different modes of sex determination Chromosome Res 2010 18 809 820 10.1007/s10577-010-9154-7 20811940

[B22] Gamble T A review of sex determining mechanisms in geckos (Gekkota: Squamata) Sex Dev 2010 4 88 103 10.1159/000289578 20234154 PMC2855288

[B23] Pokorná M Giovannotti M Kratochvíl L Kasai K Trifonov VA O'Brien PCM Caputo V Olmo E Ferguson-Smith MA Rens W Strong conservation of the bird Z chromosome in reptilian genomes is revealed by comparative painting despite 275 My divergence Chromosoma 2011 120 455 468 10.1007/s00412-011-0322-0 21725690

[B24] Jones KW Singh L Snakes and evolution of sex chromosomes Trends Genet 1985 1 55 61

[B25] O'Meally D Patel HR Stiglec R Sarre SD Geordes A Graves JAM Ezaz T Non-homologous sex chromosomes of birds and snakes share repetitive sequences Chromosome Res 2010 18 787 800 10.1007/s10577-010-9152-9 20734128

[B26] Lee MSY Hugall AF Lawson R Scanlon JD Phylogeny of snakes (Serpentes): combining morphological and molecular data in likelihood, Bayesian and parsimony analyses Syst Biodiv 2007 5 371 389 10.1017/S1477200007002290

[B27] Epplen JT McCarrey JR Sutou S Ohno S Base sequence of a cloned snake W-chromosome DNA fragment and identification of a male-specific putative mRNA in the mouse Proc Natl Acad Sci USA 1982 79 3798 802 10.1073/pnas.79.12.3798 6954524 PMC346515

[B28] Jones KW Singh L Conserved repeated DNA sequences in vertebrate sex chromosomes Hum Genet 1981 58 46 53 7286992 10.1007/BF00284148

[B29] Arnemann J Jakubiczka S Schmidtke J Schäfer R Epplen JT Clustered GATA repeats (Bkm sequences) on the human Y chromosome Hum Genet 1986 73 301 303 10.1007/BF00279090 3017838

[B30] Schäfer R Böltz E Becker A Bartels F Epplen JT The expression of the evolutionarily conserved GATA/GACA repeats in mouse tissues Chromosoma 1986 93 496 501 10.1007/BF00386790 3755389

[B31] Nanda I Feichtinger W Schmid M Schröder JH Zischler H Epplen JT Simple repetitive sequences are associated with differentiation of the sex chromosomes in the guppy fish J Mol Evol 1990 30 456 462 10.1007/BF02101117

[B32] Nanda I Zischler H Epplen C Guttenbach M Schmid M Chromosomal organization of simple repeated DNA sequences Electrophoresis 1991 12 193 203 10.1002/elps.1150120216 2040266

[B33] Parasnis AS Ramakrishna W Chowdari KV Gupta VS Ranjekar PK Microsatellite (GATA)n reveals sexspecific differences in papaya Theor Appl Genet 1999 99 1047 1052 10.1007/s001220051413

[B34] Epplen JT Cellini A Shorte M Ohno S On evolutionarily conserved simple repetitive DNA sequences: Do "sex-specific" satellite components serve any sequence dependent function? Differentiation 1983 23 Suppl S60 S63 6444177 10.1007/978-3-642-69150-8_11

[B35] Epplen JT On simple repeated GATCA sequences in animal genomes: A critical reappraisal J Hered 1988 79 409 3062083 10.1093/oxfordjournals.jhered.a110544

[B36] Ivanov VG Bogdanov OP Anislmova EY Fedorova TA Studies of the karyotypes of three lizard species (Sauria, Scincidae, Lacertidae) Tsitologiya 1973 15 1291 1296 4780025

[B37] Vidal N Hedges SB The phylogeny of squamate reptiles (lizards, snakes and amphisbaenians) inferred from nine nuclear protein-coding genes C R Biol 2005 328 1000 1008 16286089 10.1016/j.crvi.2005.10.001

[B38] Kawai A Nishida-Umehara C Ishijima J Tsuda Y Ota H Matsuda Y Different origins of bird and reptile sex chromosomes inferred from comparative mapping of chicken Z-linked genes Cytogenet Genome Res 2007 117 92 102 10.1159/000103169 17675849

[B39] Arnold AN Arrubas O Carranza S Systematics of the Palaearctic and Oriental lizard tribe Lacertini (Squamata: Lacertidae: Lacertinae), with descriptions of eight new genera Zootaxa 2007 1430 1 86

[B40] Olmo E Signorino GG Chromorep: A reptile chromosomes database http://ginux.univpm.it/scienze/chromorep/

[B41] Mayer W Pavlicev M The phylogeny of the family Lacertidae (Reptilia) based on nuclear DNA sequences: convergent adaptations to arid habitats within the subfamily Eremiainae Mol Phyl Evol 2007 44 1155 63 10.1016/j.ympev.2007.05.015 17616472

[B42] Kubat Z Hobza R Vyskot B Kejnovsky E Microsatellite accumulation on the Y chromosome in *Silene latifolia* Genome 2008 51 350 356 10.1139/G08-024 18438438

[B43] Cioffi MB Kejnovský E Bertollo LAC The chromosomal distribution of microsatellite repeats in the genome of the Wolf Fish *Hoplias malabaricus*, focusing on the sex chromosomes Cytogenet Genome Res 2011 132 289 296 10.1159/000322058 21099206

